# Chemical Modification of Phage‐Displayed Helix‐Loop‐Helix Peptides to Construct Kinase‐Focused Libraries

**DOI:** 10.1002/cbic.202100450

**Published:** 2021-10-19

**Authors:** Daisuke Fujiwara, Kousuke Mihara, Ryo Takayama, Yusuke Nakamura, Mitsuhiro Ueda, Takeshi Tsumuraya, Ikuo Fujii

**Affiliations:** ^1^ Department of Biological Science Graduate School of Science Osaka Prefecture University 1-1, Gakuen-cho, Naka-ku, Sakai Osaka 599-8531 Japan; ^2^ Department of Chemistry Graduate School of Science Osaka Prefecture University 1-1, Gakuen-cho, Naka-ku, Sakai Osaka 599-8531 Japan

**Keywords:** bivalent inhibitors, modality, peptide library, phage display, protein kinases

## Abstract

Conformationally constrained peptides hold promise as molecular tools in chemical biology and as a new modality in drug discovery. The construction and screening of a target‐focused library could be a promising approach for the generation of *de novo* ligands or inhibitors against target proteins. Here, we have prepared a protein kinase‐focused library by chemically modifying helix‐loop‐helix (HLH) peptides displayed on phage and subsequently tethered to adenosine. The library was screened against aurora kinase A (AurA). The selected HLH peptide **Bip**‐**3** retained the α‐helical structure and bound to AurA with a *K*_D_ value of 13.7 μM. **Bip**‐**3** and the adenosine‐tethered peptide **Bip**‐**3**‐**Adc** provided IC_50_ values of 103 μM and 7.7 μM, respectively, suggesting that **Bip**‐**3**‐**Adc** bivalently inhibited AurA. In addition, the selectivity of **Bip**‐**3**‐**Adc** to several protein kinases was tested, and was highest against AurA. These results demonstrate that chemical modification can enable the construction of a kinase‐focused library of phage‐displayed HLH peptides.

## Graphical abstract

**Expanding helix‐loop‐helix (HLH) peptide libraries**: A phage‐displayed HLH peptide library was chemically modified with adenosine as an anchor molecule for binding to the ATP binding site of protein kinase. This approach to the construction of target‐focused HLH peptide libraries would enable the efficient generation of selective inhibitors not only for protein kinases, but also for other proteins of interest.

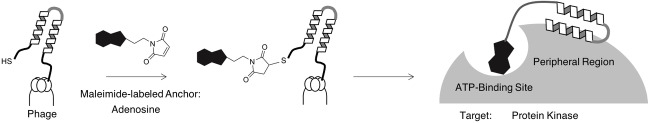

Conformationally constrained peptides binding to targeted proteins are promising powerful tools in chemical biology and drug discovery.^[1]^ Such targeting peptides have been generated by directed evolution with combinatorial libraries.^[1d]^ We previously *de novo* designed a helix‐loop‐helix (HLH) peptide and constructed phage‐ and yeast‐displayed libraries^[2]^ which were screened to afford ligands or inhibitors for ganglioside GM3, granulocyte colony stimulating factor receptor (G‐CSF‐R), cytotoxic T lymphocyte antigen‐4 (CTLA‐4), and vascular endothelial growth factor (VEGF). In addition, a cyclized HLH peptide inhibiting intracellular p53‐HDM2 interaction was generated by protein epitope grafting.^[3]^ HLH peptides are a potential new modality in molecular targeting therapy, as they display high binding activity and specificity to the target protein, and high proteolytic stability in sera. Here, we expanded our HLH peptide libraries to include a target‐focused HLH library in order to generate selective inhibitors for targeted protein kinases.

There are over 500 protein kinases in the human genome, making them an important class of enzymes.^[4]^ Since the deregulation of kinase signaling pathways often causes various diseases, a selective inhibitor for each kinase would provide therapeutic reagents. However, most small‐molecule inhibitors of kinases bind to the conserved ATP‐binding site, often non‐selectively.^[5]^ This problem has been addressed by developing bivalent inhibitors comprising two moieties: a peptide analogue of the kinase substrate, and an ATP‐competitive small compound.^[6]^ The selective‐binding peptide moiety was designed based on structural information on the target protein kinase, or by screening phage‐displayed peptide libraries. Here, we constructed a kinase‐focused library of HLH peptides by tethering an ATP‐competitive small compound to a phage‐displayed HLH peptide library (Figure 1). We chose adenosine as the ATP‐competitive small compound^[7]^ as it has relatively weak binding affinity for kinases. The adenosine part of a bivalent inhibitor screened from a focused library would weakly bind to the ATP binding site, while the HLH part would tightly interact with the kinase surface adjacent to the active site to provide affinity and selectivity for each targeted kinase.

**Figure 1:**
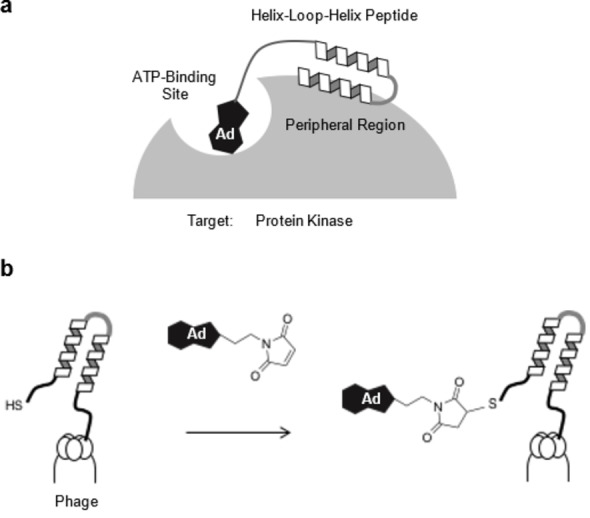
Design of target‐focused HLH peptide library directed towards protein kinase. (a) A proposed bivalent binding mode of HLH peptide tethering adenosine which binds both to the ATP‐binding site of protein kinase and to the peripheral region. (b) Schematic illustration showing chemical modification of the *N*‐terminal cysteine preceding to the HLH peptide fused with cysteine‐free pIII coat protein of filamentous phage, by using adenosine modified with maleimide.

We employed a simple maleimide‐based method to tether adenosine to phage‐displayed peptides (Figure 1b),[^8^, ^9^] in which the N‐terminal cysteine of the phage‐displayed HLH peptides was modified with adenosine containing maleimide (adenosine‐5’‐(2’‐maleimidopropionamidoethyl)‐amide (**Mal**‐**Adc**)). The adenosine derivative (**Mal**‐**Adc**) was synthesized from 2’,3′‐isopropylidene adenosine‐5′‐(2′‐aminoethyl)‐amide via reaction with maleimide‐(CH_2_)_2_‐NHS and subsequent deprotection of the isopropylidene group (Figure 2a, Supporting Scheme S1).^[10]^ The phage‐displayed HLH peptides were prepared by using the phage vector fdg3p0ss21, which encodes cysteine‐free pIII coat protein, to prevent non‐selective cysteine modification of pIII (Figure 2b, Supporting Figure S1, S2).[^8a^, ^11^] The vector was modified to produce a cysteine‐containing spacer sequence (CDGGSGGGS) upstream of the HLH peptide and the E‐tag between the HLH peptide and pIII coat protein.

**Figure 2:**
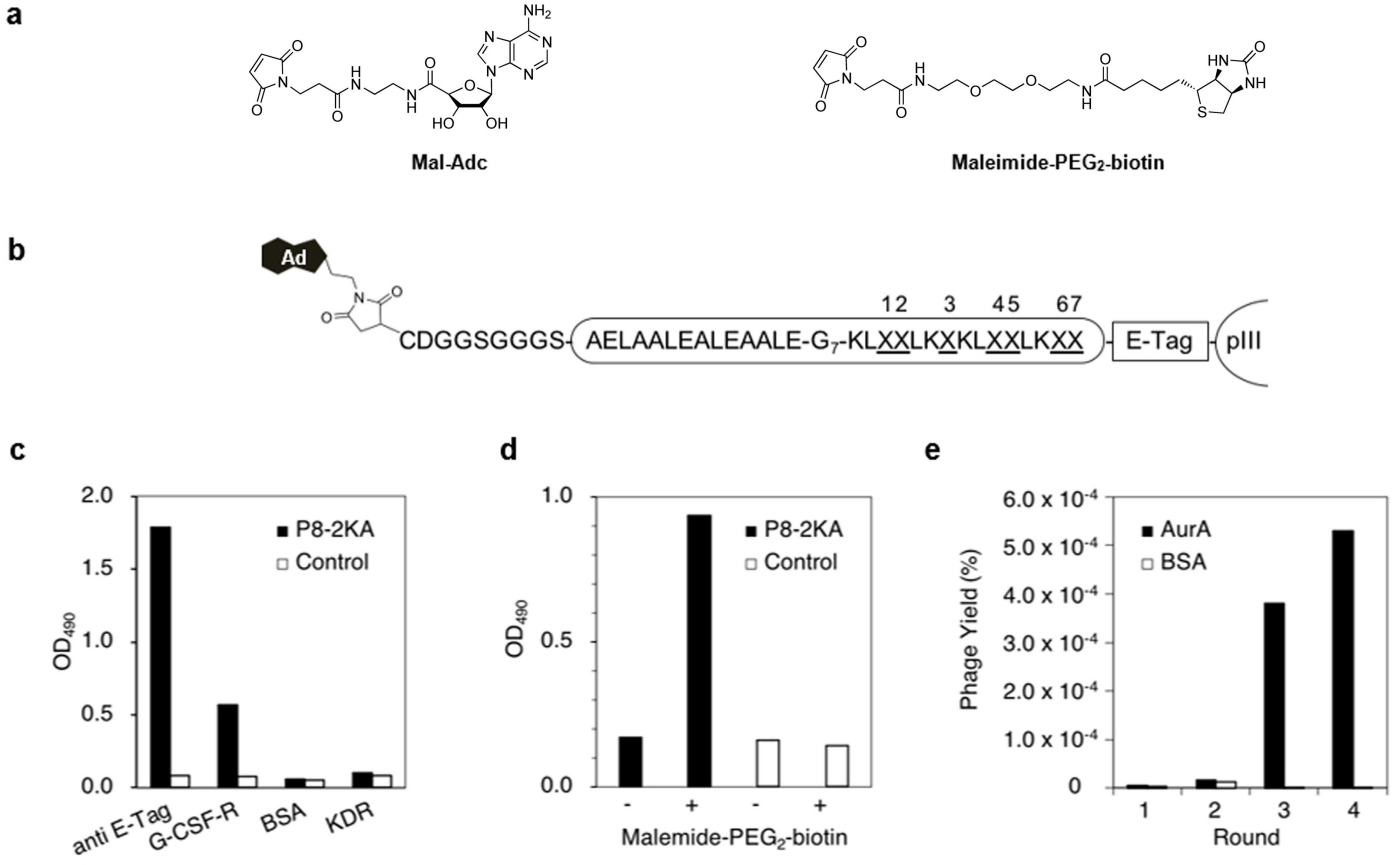
Construction of a phage‐displayed HLH peptide library, in which each peptide is tethered to adenosine, and screening against AurA. (a) The maleimide‐containing compounds used in this study. Maleimide‐modified adenosine (**Mal**‐**Adc**) and maleimide‐PEG_2_‐biotin. (b) The prepared phage‐displayed HLH peptide library treated with **Mal**‐**Adc**. X represents positions of randomly mutated amino acids and G_7_ represents a 7‐mer glycine linker. (c) Phage ELISA experiments to confirm the display of HLH peptides on the phage surface. Phage clones displaying peptide **P8**‐**2KA** and control clones displaying neither the peptide nor E‐tag were prepared. The four proteins anti‐E Tag antibody, G‐CSF‐R, BSA, and KDR were immobilized on an ELISA plate. The binding clones were detected by using anti‐phage antibody‐HRP conjugate. (d) Phage ELISA using a streptavidin‐coated microtiter plate. Phage clones were treated with 200 μL of 5 μM maleimide‐PEG_2_‐biotin at room temperature for 2 h. (e) Enrichment of phage clones selectively binding to AurA after each round of bio‐panning using the HLH peptide library tethering adenosine.

Prior to constructing the kinase‐focused HLH phage library, we examined the reaction conditions for chemical modification of HLH peptides displayed on the phage surface. Mild conditions are required for modification to minimize damage to phage clones so that they remain infectious to the host *Escherichia coli* cells.^[8a]^ A phage clone displaying the HLH peptide **P8**‐**2KA** (CDGGSGGGS‐AELAALEAELAALE‐G_7_‐KLAMLKLKLAELKRY), used as a model, binds to G‐CSF‐R with a *K*_D_ value of 214 nM.^[2e]^ As shown in Figure 2c, the phage clone bound to anti‐E‐tag antibody, confirming peptide display on the phage surface. The selective binding of **P8**‐**2KA** to G‐CSF‐R demonstrated its specificity; no binding was observed to the two other proteins examined, BSA and KDR (VEGF receptor). Phage clones (10^11^ cfu) of **P8**‐**2KA** were treated with 200 μL of serially diluted maleimide‐PEG_2_‐biotin (0.5–500 μM) in PBS at room temperature for 2 h (Supporting Figure S3). Higher concentrations of maleimide‐PEG_2_‐biotin led to a small loss of phage infectivity of the host TG1 *E. coli* cells and thus in further experiments we chemically modified the phage clones with 5 μM maleimide‐PEG_2_‐biotin. In phage ELISA experiments (Figure 2d), phage clones modified with PEG_2_‐biotin were detected by their binding to streptavidin‐immobilized microtiter plates whereas the negative control phage showed no binding to streptavidin.

We used the *de novo* designed HLH peptide **YT1** (AELAALEAELAALE‐G_7_‐KLAALKAKLAALKAY)^[2]^ to guide the construction of a phage‐displayed library in which each peptide tethered an adenosine. Seven alanine residues in the C‐terminal helix were randomly mutated (Figure 2b). Freshly prepared phage clones displaying the HLH peptide library were treated with **Mal**‐**Adc** using the conditions described above. The chemically modified phage library was screened against AurA, a serine/threonine protein kinase involved in regulating mitosis and a common target of anti‐cancer drug discovery.^[12]^ Phage clones binding to AurA were enriched after four rounds of bio‐panning whereas no enrichment was observed in screening against the control protein BSA (Figure 2e). Sixteen clones were randomly selected and the HLH peptide amino acid sequences were determined (Supporting Table 2, Supporting Figure S4). Peptide **Bip**‐**3** (CDGGSGGGS‐AELAALEAELAALE‐G_7_‐KLEYLKWKLWPLKG W) was most frequently observed. Interestingly, **Bip**‐**3** showed no sequence homology with known ligands and inhibitors for AurA.^[13]^ We synthesized **Bip**‐**3** using a standard solid‐phase method and examined it in detail.

CD spectrometry revealed that **Bip**‐**3** has a high α‐helical content comparable with that of the original peptide **YT1** (Figure 3b).^[2g]^ Surface plasmon resonance (SPR) experiments provided a *K*_D_ value of 13.7 μM for **Bip**‐**3** using AurA immobilized on a sensor chip (Figure 3c). The inhibitory activity of the peptide for AurA was determined using an IMAP® TR‐FRET assay.^[14]^ Peptide **Bip**‐**3** showed an IC_50_ value of 103 μM (Figure 3d) whereas the control **YT1** showed no inhibitory activity. As shown in Figure 1a, a kinase‐targeting peptide screened from our peptide library would bind to a targeted peripheral region of the ATP binding site. Therefore, to examine the competitive binding mode to ATP, the inhibition activities of peptide **Bip**‐**3**, adenosine, and the inhibitor **VX**‐**680** (designed to target the ATP binding site of AurA) were measured using various concentrations of ATP.[^15^, ^16^] Lineweaver‐Burk plot analysis showed that adenosine and **VX**‐**680** exhibited competitive inhibition for ATP whereas **Bip**‐**3** showed mixed inhibition (Supporting Figure S5). Mixed‐type inhibition occurs, by definition, when an inhibitor binds the enzyme both before and after substrate binding and the affinities for the free enzyme and ES complex are different.^[17]^

**Figure 3:**
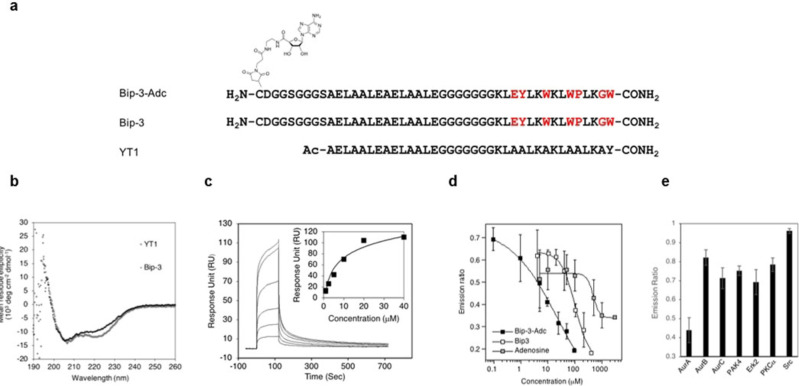
Inhibitory activities of the selected peptide **Bip**‐**3** and the adenosine‐tethering peptide **Bip**‐**3**‐**Adc** for AurA. (a) The structure of synthesized **Bip**‐**3**‐**Adc**, **Bip**‐**3**, and the original HLH peptide **YT1**. (b) The CD spectra of 20 μM **Bip**‐**3** and **YT1** measured in TBS at 20 °C. (c) The binding activity of **Bip**‐**3** to immobilized AurA as determined by SPR experiments (TBS, 0.005 % P‐20, 25 °C). (d) Dose‐dependent inhibitory activities of **Bip**‐**3** and **Bip**‐**3**‐**Adc** for AurA measured using an IMAP® TR‐FRET assay. Inhibition of AurA was determined at 100 ng/mL AurA, 1 mM 5FAM‐Kemptide, 5 mM ATP, in TBS reaction buffer. (e) Inhibitory activities of 5 μM **Bip**‐**3**‐**Adc** for AurA, AurB, AurC, PAK4, Erk2, PKACα, and Src, determined using the IMAP® TR‐FRET assay.

Next, peptide **Bip**‐**3** was chemically modified with **Mal**‐**Adc** to provide the adenosine‐tethering peptide **Bip**‐**3**‐**Adc**, which was subjected to an IMAP^®^ TR‐FRET inhibition assay for AurA (Figure 3a). **Bip**‐**3**‐**Adc** inhibited AurA with an IC_50_ value of 7.7 μM, which is 13‐fold higher than that of **Bip**‐**3** (Figure 3d) and 62‐fold higher than of adenosine (IC_50_: 474 μM). This result suggested that **Bip**‐**3**‐**Adc** interacted with the ATP‐binding pocket and its peripheral region, and the peptide moiety **Bip‐3** appeared to dominantly contributed to the inhibitory activity for AurA. Lineweaver‐Burk plot analysis showed that **Bip**‐**3**‐**Adc** exhibited uncompetitive inhibition (Supporting Figure S5).^[18]^ Future studies will aim to clarify the mode of this bivalent inhibition of **Bip**‐**3**‐**Adc** and obtain structural information. Finally, we evaluated the selectivity of **Bip**‐**3**‐**Adc** for AurA by comparing its inhibition activity against the aurora protein kinases AurA, AurB, and AurC, the serine/threonine‐protein kinases PAK4, Erk2, and PKAcα, and the tyrosine‐protein kinase Src. As shown in Figure 3e, **Bip**‐**3**‐**Adc** exhibited the highest inhibitory activity and remarkable selectivity for AurA.

In addition to using the kinase‐focused HLH peptide library, we attempted to generate AurA‐kinase inhibitors by screening several phage libraries: commercially available libraries of linear 7‐ (X_7_) and 12‐mer (X_12_) peptides, 7‐mer cyclic peptides with a disulfide bond (CX_7_C), and our conventional ‘α‐helical’ library (AELAALEAELAALE‐G_7_‐KLXXLKXKLXXLKA). Unfortunately, we observed no enrichment of phage clones binding to AurA. Screening of our 9‐mer ‘loop’ library **L**‐**lib11** (AELAALEAELAALE‐GX_9_G‐KLAALKAKLAALKA) gave an HLH peptide inhibitor against AurA, but it had only weak inhibitory activity, showing 35 % inhibition at a concentration of 100 μM.^[2d]^ Given these results, we concluded that the newly constructed kinase‐focused HLH library is a powerful tool for the efficient generation of *de novo* kinase inhibitors.

In summary, in this work we constructed a phage‐displayed library of HLH peptides tethered to adenosine to generate a selective inhibitor for AurA. Screening of this library identified the binding peptide **Bip**‐**3**, which was subsequently tethered with adenosine to generate the bivalent inhibitor **Bip**‐**3**‐**Adc**. As expected, **Bip**‐**3**‐**Adc** showed remarkable selectivity for AurA. In future, **Bip**‐**3**‐**Adc** could be developed as a molecular tool for chemical biology and as a therapeutic lead for cancer therapy by further affinity maturation and by making it cell‐membrane permeable.[^3^, ^19^] Furthermore, use of a different tethered anchor compound would enable the construction of a variety of HLH peptide libraries targeting various proteins of interest. This approach to the construction of target‐focused HLH peptide libraries would enable the efficient generation of selective inhibitors not only for protein kinases, but also for other proteins of interest.

## Experimental Section

See the Supporting Information for full details.

## Conflict of interest

The authors declare no conflict of interest.

## Supporting information

As a service to our authors and readers, this journal provides supporting information supplied by the authors. Such materials are peer reviewed and may be re‐organized for online delivery, but are not copy‐edited or typeset. Technical support issues arising from supporting information (other than missing files) should be addressed to the authors.

Supporting Information
