# Supplementary material for: Chemical Modification of Phage‐Displayed Helix‐Loop‐Helix Peptides to Construct Kinase‐Focused Libraries
Source: Chembiochem. 2021 Oct 19;22(24):3406–9. doi: 10.1002/cbic.202100450 (PMC9297947; doi:10.1002/cbic.202100450)
Supplement: Supplementary file 1 — Supporting Information [file CBIC-22-3406-s001.pdf]

ChemBioChem

Supporting Information

Chemical Modification of Phage-Displayed Helix-Loop-Helix Peptides to Construct Kinase-Focused Libraries

Daisuke Fujiwara, Kousuke Mihara, Ryo Takayama, Yusuke Nakamura, Mitsuhiro Ueda, Takeshi Tsumuraya, and Ikuo Fujii*

Table of Contents

1. Helix-Loop-Helix peptide displayed on filamentous phages

2. Chemical Modification of HLH peptide on Phage

3. The Infectivity of phages after Chemical Modification

4. Synthesis of Maleimide-labeled Adenosine

5. The kinase activity of immobilized Aurora kinase A

6. Affinity Selection with Aurora kinase A

7. Peptide Synthesis

8. Circular Dichroism

9. Surface Plasmon Resonance

10. IMAP® TR-FRET Assay

11. Inhibition Mechanism of Peptide inhibitor Bip-3 for Aurora kinase A

1. Helix-Loop-Helix peptide displayed on filamentous phages

All of the experiments were performed as described [1,2]. In this study, the insert fragment encoding of helix-loop-helix (HLH) peptide **P8-2KA** and phage clone displaying no peptide were prepared. With regard to clone **P8-2KA**, the insert fragment was initially prepared by overlap extension PCR using **P8-2KA 1 fwd** and **P8-2KA 1 rev**. After purification of the fragment, restriction site *Sfi*I and E-tag epitope were introduced by the following PCR using **Sfi-YT1-CD 2 fwd** and **Etag-linker 2 rev**, and the product was purified. The fragment was fused with the pIII gene amplified by the two primers **prepcr** and **Sfi2fo** with the template fdg3p0ss21, and a fragment encoding P8-2KA-fused pIII was prepared as described. After digestion of the fragment by *Sfi*I and the succeeding purification, the fragment was ligated with fdg3p0ss21-stop which was digested with the same restriction enzyme. As a negative control, we also prepared a clone displaying no HLH peptide on phage. To prepare the phage clone, **only linker primer** was used in the PCR together with Sfi2fo and the pIII gene amplified from the template fdg3p0ss21 as described above. The titers of the prepared phage clones were 3.6 x 10^{12} cfu/mL for **P8-2KA** and 7.5 x 10^{12} cfu/mL for the phage displaying no HLH peptide. With regard to the peptide library, the initial overlap extension PCR was performed by using **7lib 1 fo** and **7 lib 1 ba**, and the succeeding experiments were performed likewise. The diversity of the library was estimated as 1.0 x 10^{7}, and with the titer of 4.6 x 10^{12} cfu/mL. The varied sequences of HLH peptides in the library were confirmed by DNA sequencing.

To confirm display of HLH peptide on phage, we performed ELISA experiments. Fifty μL of diluted Anti-Etag antibody (1:1000), 2 ng/μL Granulocyte colony-stimulating factor receptor (G-CSF-R) [3] , 2 ng/μL Fc-fused Vascular endothelial growth factor receptor (KDR), and 2% BSA were respectively immobilized on 96-well ELISA plates. After washing wells with PBS containing 0.05% Tween 20 (PBST) 4 times, the wells were blocked with SuperBlock® Blocking Buffer in PBS (Thermo Scientific) for 1 hr at 4°C. Phage solution (10^{10} cfu) was added to the wells, and incubated for 1 hr with shaking at room temperature. After washing the wells with PBST 10 times, 100 μL of HRP Anti-M13 antibody conjugated (1:4000) was added, and the OD_{490} values were measured after adding *o*-phenylene diamine (SIGMA*FAST*™ OPD, SIGMA) and adding 50 μL of 2 N H_2SO_4.

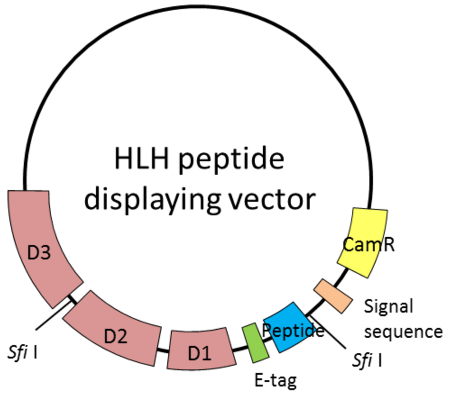

Figure S1. Phage vector displaying HLH peptide

Table S1. Oligonucleotides to construct phage-displayed HLH peptide and the libraries.

No.	Oligonucleotide sequence
**P8-2KA 1 fwd**	GCGGCACTCGAAGGTGGCGGCGGTGGTGGCGGCAAGCTGGCTATGTTGAAACTCAAGCTGGTTGAACTGA
**P8-2KA 1 rev**	GCACCGGCGCACCTGCGCTCGAGGAACCGCCTCCGGAGCCTCCACCATAACGTTTCAGTTCAACCAGCTT
**Sfi-YT1-CD 2 fwd**	TATGCGGCCCAGCCGGCCATGGCATGCGACGGTGGCAGTGGAGGTGGCTCCGCCGAACTGGCAGCTCTGGAA GCGGAACTGGCGGCACTCGAAGGTG
**Etag-linker 2 rev**	CAGCGCCGGATCCGCCACTAGTACGCGGTTCCAGCGGATCCGGATACGGCACCGGCGCACCTGCGCTCGA
**only linker primer**	TATGCGGCCCAGCCGGCCATGGCAGGTGGAGGCTCCGGCGGATCCGGCGCTG
**7lib 1 fo**	GCACTCGAAGGTGGCGGCGGTGGTGGCGGCAAGCTGNNKNNKTTGAAANNKAAGCTGNNKNNKCTGAAAN NKNNKGGTGGAGGCT
**7lib 1 ba**	ACGGCACCGGCGCACCTGCGCTCGAGGAACCGCCTCCGGAGCCTCCACC

N stands for A, C, G, and T. K stands for G and T.

```
A  A  Q  P  A  M  A  C  D  G  G  S  G  G  G  S  A  E  L  A
GCGGCCCAGCCGGCCATGGCATGCGACGGTGGCAGTGGAGGTGGCTCCGCCGAACTGGCA
  SfiI

A  L  E  A  E  L  A  A  L  E  G  G  G  G  G  G  G  K  L  X
GCTCTGGAAGCGGAACTGGCGGCACTCGAAGGTGGCGGCGGTGGTGGCGGCAAGCTGNNK

X  L  K  X  K  L  X  X  L  K  X  X  G  G  G  S  G  G  G  S
NNKTTGAAANNKAAGCTGNNKNNKCTGAAANNKNNKGGTGGAGGCTCCGGAGGCGGTTCC

S  S  A  G  A  P  V  P  Y  P  D  P  L  E  P  R  T  S  G  G
TCGAGCGCAGGTGCGCCGGTGCCGTATCCGGATCCGCTGGAACCGCGTACTAGTGGCGGA

S  G  A  E  T  V  E  S  S  L  A  K  S  H  I  E  G  S  F  T
TCCGGCGCTGAAACTGTTGAAAGTAGTTTAGCAAAATCCCATATAGAAGGTTCATTTACT

N  V  W  K  D  D  K  T  L  D  W  Y  A  N  Y  E  G  I  L  W
AACGTCTGGAAAGACGACAAAACTTTAGATTGGTACGCTAACTATGAGGGCATCCTGTGG

K  A  T  G  V  V  V  I  T  G  D  E  T  Q  V  Y  A  T  W  V
AAGGCTACAGGCGTTGTAGTTATAACTGGTGACGAAACTCAGGTATACGCTACATGGGTT

P  I  G  L  A  I  P  E  N  E  G  G  G  S  E  G  G  G  S  E
CCTATTGGGCTTGCTATCCCTGAAAATGAGGGTGGTGGCTCTGAGGGTGGCGGTTCTGAG

G  G  G  S  E  G  G  G  T  K  P  P  E  Y  G  D  T  P  I  P
GGTGGCGGTTCTGAGGGTGGCGGTACTAAACCTCCTGAGTACGGTGATACACCTATTCCG
```

Orange:Signal sequence

Blue:Peptide library

Green:E-tag sequence

Red:D1 of pIII

Brown:D2 of pIII

Black:Linker

Figure S2. The HLH peptide library to tether adenosine.

2. Chemical Modification of HLH peptide on Phage

All of the experiments were performed as described [4]. 200 μL of phage (10^{12} cfu) was dissolved in PBS (pH 7.0), and 1 μL of 1 mM Maleimide-PEG$_2$-biotin (EZ-Link® Maleimide-PEG$_2$-Biotin, Thermo Scientific) was added and incubated for 2 hr at room temperature. To eliminate the residual reagents in phage solution, the treated solution was purified by ultrafiltration at 14,000 g for 20 min at 4 C by using Amicon® Ultra 0.5 mL Ultracel® 10K (Millipore). After discarding the flow-through, 200 μL of chilled PBST was added, and centrifugated as the same above. 100 μL of the chemically modified phage was added to Streptavidin High Capacity Coated Plate (SIGMA). Detection of phages was performed as described above by using Anti-M13 antibody conjugated with HRP.

3. The Infectivity of phages after Chemical Modification

All of the experiments were performed as described [5]. One μL of serially diluted Maleimide-PEG$_2$-biotin from 100 μM to 100 mM was added to 200 μL of the phage clone of **P8-2KA** (10^{12} cfu) and incubated for 2 hr, and centrifugated as described above. *E. coli* TG1 was cultured in 3 mL SB medium at the OD_{600} 0.3–0.5, and the phage solution was added to the culture and incubated for 90 min at 37°C. Then the *E. coli* cells were inoculated onto LB/Agar containing 20 μg/mL chloramphenicol over night at 37°C, and colonies were counted.

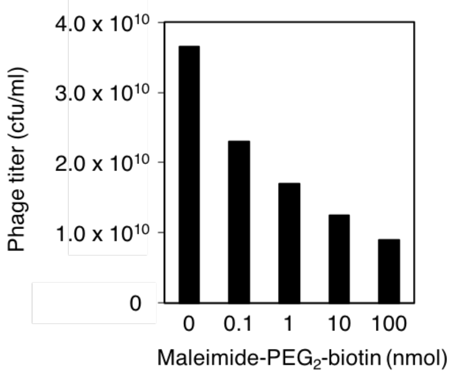

Figure S3. Titers of phage treated with Maleimide-PEG$_2$-biotin as in the method.

4. Synthesis of Maleimide-labeled Adenosine

We synthesized adenosine-5’-(2’-maleimidopropionamidoethyl)-amide (Mal-Adc) by following the synthetic route of adenosine derivatives [6, 7]. ^{1}H and ^{13}C NMR spectra were obtained on a JEOL ECS-400 spectrometer. High-resolution mass spectra (HRMS) were recorded on a Bruker micrOTOF II mass spectrometer.

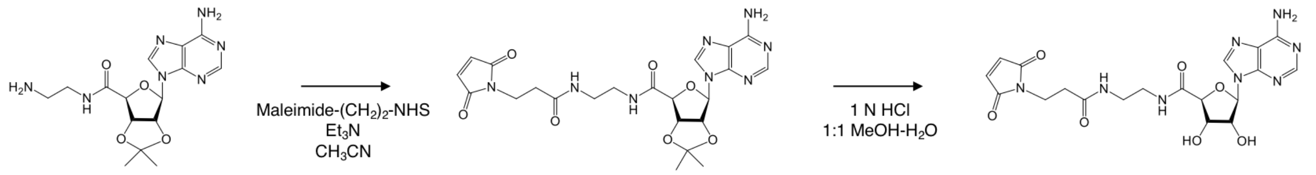

1 2 3

Scheme S1. Synthesis of maleimide-labeled adenosine.

2’,3'-*O*-Isopropylideneadenosine-5'-(2'-maleimidopropionamidoethyl)-amide (2)

2’,3'-*O*-Isopropylideneadenosine-5'-(2'-aminoethyl)-amide (Santa Cruz, sc-206552) (**1**) (68.2 mg, 0.19 mmol) was dissolved in 3 mL of acetonitrile (ACN), and triethylamine (52 µl, 0.38 mmol) was added and stirred. *N*-(3-Maleimidopropionyloxy)succinimide (100 mg, 0.375 mmol) was added to the mixture and stirred for 3 h. The solution was distilled off under reduced pressure, and the residue was purified by HPLC (Water, 0.1%TFA /ACN 18/82). Elution was performed by Hitachi HPLC with C18 reversed-phase column (YMC) (250 mm × 10 mm inside diameter (I.D.), 5 µm), with monitoring at 260 nm with a flow rate of 3 mL/min. The eluate was dried to give 2 (21.6 mg, 22%). ^{1}H-NMR (400 MHz, DMSO-d$_6$) : δ 8.42 (s, 1H), 8.24 (s, 1H), 7.87 (s, 1H), 7.84 (s, 1H), 7.05 (s, 1H), 6.99 (s, 2H), 6.36 (s, 1H), 5.36 (s, 2H), 4.59 (s, 1H), 3.74 (t, *J* = 6.8 Hz, 2H), 3.55 (t, *J* = 7.2 Hz, 2H), 3.04 (t, J = 7.3 Hz, 2H), 2.78 (s, 2H), 2.25 (t, *J* = 7.3 Hz, 2H), 1.54 (s, 3H), 1.34 (s, 3H); ^{13}C-NMR (125 MHz, DMSO-d$_6$): δ 171.2, 170.1, 169.2, 167.0, 148.9, 142.5, 135.1, 131.8,119.2, 117.0, 113.6, 90.4, 86.6, 83.8, 83.7, 65.6, 38.7, 38.2, 34.5, 34.5, 27.2, 25.5; HRMS (ESI$^+$): m/z calcd for $C_{22}H_{27}N_8O_7$ [M+H]$^+$, 515.1997; found, 515.2003.

Adenosine-5'-(2'-maleimidopropionamidoethyl)-amide (3)

Compound **2** (15.6 mg, 33 µmol) was dissolved in 3 mL of MeOH/Water (1/1) and 50 µL of 1 N HCl was added and stirred for 24 hr at 55°C. The solution was purified by HPLC (Water, 0.1%TFA /ACN 10/90) with a flow rate of 3.0 mL / min with monitoring 260 nm. The eluate was dried to give **3** (6.1 mg, 9 %) as a white powder. ^{1}H-NMR (400 MHz, DMSO-d$_6$) : δ 8.51 (s, 1H), 8.31 (s, 1H), 8.07 (s, 1H), 6.99 (s, 2H), 5.98 (d, *J* = 7.8, 1H), 4.59 (dd, *J* = 4.5 and 2.9, 1H), 4.32 (d, *J* = 1.2 Hz, 1H), 4.18 (d, *J* = 4.1 Hz, 1H), 2.78 (t, *J* = 7.3, 2H), 3.07-3.22 (m, 3H), 2.28 (t, *J* = 7.3 Hz, 2H); ^{13}C-NMR (125 MHz, DMSO-d$_6$): δ 215.2, 171.3, 170.2, 170.1, 158.5, 149.1, 146.0, 142.2, 135.1, 119.8, 88.3, 85.2, 73.6, 73.1, 49.6, 38.8, 38.7, 34.7, 34.5; HRMS (ESI$^+$): m/z calcd for $C_{19}H_{23}N_8O_7$ [M+H]$^+$, 475.1684; found, 475.1668.

5. The kinase activity of immobilized Aurora kinase A

GST-tagged Aurora kinase A (GST-AurA) (Carna Bioscience), autophosphorylated in advance, was immobilized onto ELISA plate (Polysorp, NUNC) and retention of the kinase activity was confirmed (Figure S4). 50 μL of diluted (1:1000) anti-GST antibody (Amersham Biosciences) solution was incubated over night at 4℃ on the plates. After washing the wells four times with chilled 50 mM Tris-HCl buffer (pH 7.5), 150 mM NaCl, 0.02% Tween 20, 300 μL of chilled blocking reagent, 2% BSA, was added and incubated for 1 h at 4℃. After washing the wells, 50 μL of GST- AurA was incubated in the wells for 1 hr. Thirty μL of reaction mixture including 5-FAM PKAtide and ATP was added to each well, and reacted for 1 hr in dark. After transferring 15 μL of solutions to 384-well white plate (Greiner Bio-One), the kinase activity was detected by IMAP® TR-FRET (Molecular Devices) with Varioskan Flash (Thermo Scientific) [8-10].

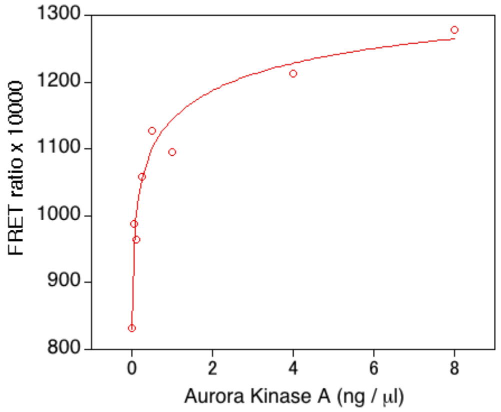

Figure S4. The kinase activity of immobilized AurA.

6. Affinity Selection with Aurora kinase A

GST-tagged aurora kinase A (AurA) was purchased from Carna Biosciences. The screening procedures are performed as described [1, 2, 11]. Here we used adenosine-labeled HLH peptide library as an input phage for the 1st round of bio-panning against AurA, while BSA-coated well was used as a negative control for monitoring selective enrichment of phages binding to AurA. Freshly prepared phages from output phage were treated with Mal-Adc at every round, and added to AurA immobilized microtiter wells. We randomly picked up 16 clones from the output phage in round 4 for AurA. The deduced amino acid sequences of the peptides encoded by the clones were determined by DNA sequences analyzed with 3100-Avant Genetic Analyzer (Applied Biosystems). The WebLogo representing the pattern within a multiple alignment was generated from the deduced amino acids (Figure S5) [12] .

Table S2. Deduced amino acid sequences of the selected peptides encoded by the output phages in round 4.

Clone	Deduced amino acid sequences	Frequency
**Position No. for WebLogo**	----------------------12--3--45--67	
**α-helical library**	AELAALEAELAALEGGGGGGGKLXXLKXKLXXLKXX	
**Bip-3**	----------------------EY--W--WP--GW	3/16
**Bip-4**	----------------------HV--D--LP--GG	2/16
**Bip-1**	----------------------ER--A--LD--YM	1/16
**Bip-2**	----------------------GG--W--WP--GW	1/16
**Bip-6**	----------------------VN--S--WP--EW	1/16
**Bip-7**[a]	----------------------*G--M--WP--DW	1/16
**Bip-8**	----------------------HT--S--IP--RE	1/16
**Bip-9**[b]	----------------------ET- -WP--GW	1/16
**Bip-12**	----------------------GL--W--PI--QD	1/16
**Bip-13**	----------------------PL--W--PV--SS	1/16
**Bip-14**	----------------------QI--W--PV--GD	1/16
**Bip-15**[a]	----------------------RN--F--G*--EG	1/16
**Bip-16**[a]	----------------------PG--*--AW--WR	1/16

[a] Amber codon (*) could be translated into Glutamine (Q) by amber repressor sup*E* gene of *E. coli* TG1.
[b] Space: Deletion mutation

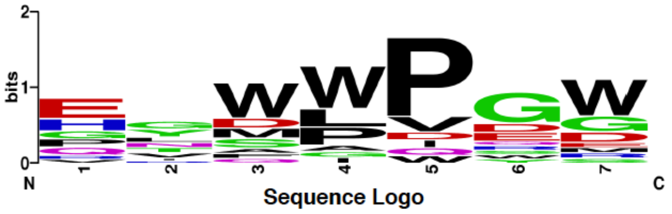

Figure S5. WebLogo of the seven positions of the selected HLH peptides against AurA.

7. Peptide Synthesis

Peptides were synthesized by standard solid-phase peptide synthesis (SPPS) with Fmoc amino acids [13], by using an automated solid phase peptide synthesizer (CS336X, CS Bio). 0.1 mmol of Fmoc-NH-SAL-PEG resin was deprotected with 20% piperidine/DMF (v/v) and washed with DMF. 0.5 mmol of Fmoc-Amino acid was activated with HBTU or HCTU and added to the deprotected Fmoc amide resin. The subsequent elongation of peptides was performed in the same way, and the synthesized peptide was cleaved with TFA containing scavengers. The cleaved peptide was filtered and the flow-through was centrifuged in chilled diethyl ether, and then dried. The peptides were purified with reversed-phase high-performance liquid chromatography (RP-HPLC) (C18 column, 250 mm x 10 mm I.D., 5 µm, YMC) with a linear gradient of water containing 0.1% TFA and acetonitrile at a flow rate 3 mL/min by using HPLC system (Hitachi). The synthesized peptides were identified by matrix-assisted laser desorption ionization time-of-flight mass spectrometry (MALDI-TOF-MS) (Autoflex II, Bruker Daltonics) using CCA in acetonitrile/0.1% (v/v) TFA (1:2) as a matrix. The peptide purities were determined with analytical HPLC (C18 column, 250 mm x 4.6 mm I.D., 5 µm, YMC), and verified over 90% in all peptides.

Mal-Adc was reacted with **Bip-3** in PBS (pH 7.0) for 6 hr at room temperature, and **Bip-3-Adc** was purified by RP-HPLC as described above. MALDI TOF-MS (m/z) **Bip-3**: Calcd.: 44457.251 $[M+H]^+$; Found 4457.370. $[M+H]^+$. MALDI TOF-MS (m/z) **Bip-3-Adc**: Calcd $[M+H]^+$: 4930.681; Obs (m/z) 4930.860.

8. Circular Dichroism

CD spectra were recorded in TBS at overall peptide concentration of 20 μM on a J-820 spectrometer at 20°C (PTC-423L thermostat, Jasco). The peptide concentrations were determined by measuring absorbance of aromatic residues of peptide dissolved in 6 M Gdn•HCl at UV 280 nm[14].

9. Surface Plasmon Resonance

SPR measurements were carried out with Biacore T200 instrument [15]. In direct binding experiments, 25 μg/mL GST-tagged AurA (Carna Biosciences, Inc.) dissolved in 10 mM sodium borate (pH 7.0) was immobilized on a CM5 sensor chip by amine coupling method (8735 resonance units (RUs)) using HBS-EP as running buffer, and blocked with ethanolamine; a reference cell was also blocked with ethanolamine. To avoid denaturation of AurA, measurements were performed without regeneration of the sensor chip surface at each cycle, and any cycles were started after complete dissociation of analytes from the sensor chip. Binding assays were carried out at 25°C using TBS, 0.005% P-20 as a running buffer, and the K_D values of peptides were determined by fitting 1:1 Langmuir binding model to the data. The concentration of adenosine-labeled peptide was determined by using the molar extinction coefficient of adenosine [16], and aromatic residues at UV 260 nm. As a positive control, the K_D value of Staurosporine was estimated to be 5.6 nM in the condition using TBS, 0.005% P-20, 5% DMSO as a running buffer, with DMSO solvent correction.

10. IMAP® TR-FRET Assay

5-FAM labeled peptide substrates were synthesized by standard SPPS peptide synthesis, and used in IMAP® TR-FRET assays [17, 18]. Each peptide was labeled with 5-carboxyfluorescein at the N-terminal amine group on resin, cleaved, and purified by RP-HPLC. All of the GST-tagged protein kinases were purchased from Carna Bioscience. Two μL of protein kinase, 2 μL of substrate/ATP, and 2 μL of serially diluted peptide solutions were incubated for 1 hr at room temperature, and 18 μL of binding buffer containing binding reagent was added to the reaction solutions. TR-FRET signals were detected by using with Varioskan Flash (Thermo Scientific), and corrected/Tb Rfu x 10,000 were plotted as described. Optimized assay conditions following the guides are as follows (Table S2).

Table S2. Assay conditions of IMAP® TR-FRET for the respective protein kinases

Protein Kinase	Concentration (ng/mL)	ATP (μM)	Peptide Substrate		
			Name	Sequence	(μM)
AurA	100	5	FAM-Kemptide	FAM-LRRASLG-OH	1
AurB	75	5	FAM-Kemptide	FAM-LRRASLG-OH	1
AurC	240	5	FAM-Kemptide	FAM-LRRASLG-OH	1
PAK4	600	5	FAM-PAK4 substrate	FAM-KKRNRRLSVA-OH	1
Erk2	40	10	FAM-Erktide	FAM-ATGPLSPGPFGRR-NH_2	1
PKAcα	8	5	FAM-Kemptide	FAM-LRRASLG-OH	1
Src	30	10	FAM-Srctide	FAM-AEEEIYGEFEAKKKK-NH_2	1

11. Inhibition Mechanism of Peptide inhibitor Bip-3 for Aurora-A

To provide insight on how **Bip-3** inhibited AurA, we examined the effects of increasing concentrations of ATP on peptide inhibition. Lineweaver-Burk plots were generated by plotting the TR-FRET ratio values, which are proportional to reaction velocity in these conditions [8,9], as a function of the velocity versus ATP concentration ratio (v/[ATP]) at different concentrations of the peptide [19,20]. Adenosine and an AurA-selecive inhibitor VX-680 (EpiGentek) showed ATP-competitive inhibitory activities for AurA. [21]

$$1/\text{FRET ratio} = K_m/(\text{FRET ratio max}*[S])+1/\text{FRET ratio max}$$

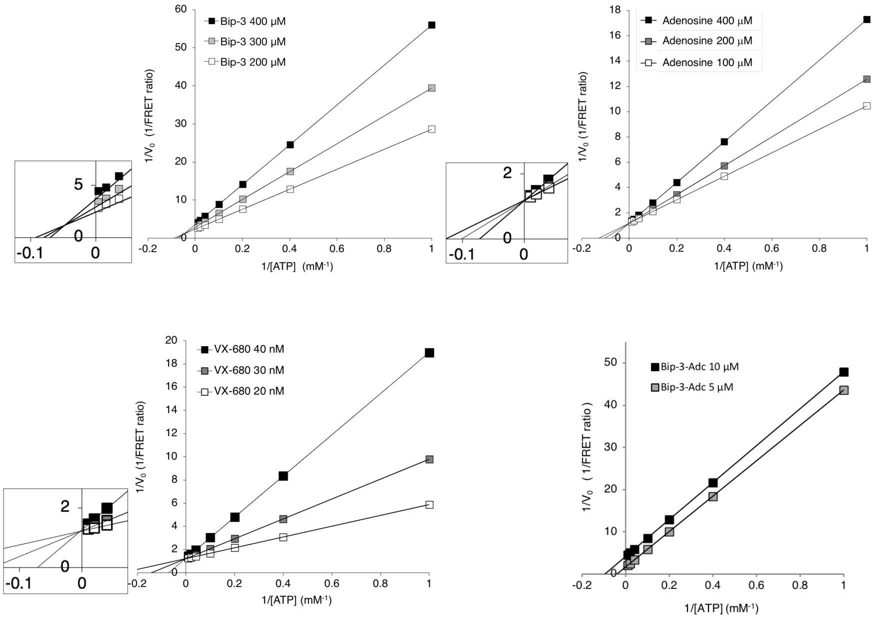

Figure S6. Inhibitory mechanisms of **Bip-3**, adenosine, **VX-680**, and **Bip-3-Adc** for AurA.

References

[1] S. Chen S, C. Heinis, C*Methods Mol Biol.* **2015**, *1248*, 119–137.

[2] K. Arai K, H. Tsutsumi, Mihara, *Bioorg. Med. Chem. Lett.* **2013**, 23, 4940–4943.

[3] R. El-Haggar, K. Kamikawa, K. Machi, Z. Ye, Y. Ishino, T. Tsumuraya, I. Fujii, *Bioorg. Med. Chem. Lett.* **2010**, *20*, 1169–1172.

[4] B. Santoso, B.W. Murray, *Methods Mol. Biol.* **2015**, *1248*, 267–276.

[5] C. Heinis, T. Rutherford, S. Freund, G. Winter, *Nat. Chem. Biol.* **2009**, *5*, 502–507.

[6] J. B. Epp, T. S. Widlanski, *J. Org. Chem.* **1999**, *64*, 293–295.

[7] K. A. Jacobson, M. Ohno, H. T. Duong, S. K. Kim, S. Tchilibon, M. Česnek, A. Holý, Z. G. Gao, *Chem. Biol.* **2005**, *12*, 237–247.

[8] E.R. Sharlow, S. Leimgruber, T.Y. Shun, J.S. Lazo, *Assay Drug Dev. Technol.* **2007**, *5*, 723–735.

[9] E.R. Sharlow, S. Leimgruber, A.Yellow-Duke, R. Barrett,. Q.J. Wang, J.S. Lazo, *Nat. Protoc.* **2008**, *3*, 1350–1363.

[10] K.S. Oh, J. Mun, J.E. Cho, S. Lee, K.Y. Yi, C.J. Lim, J.S. Lee, W.J. Park, B.H. Lee, *Comb. Chem. High Throughput Screen.* **2013**, *16*, 37–46.

[11] D. Fujiwara, I. Fujii, *Curr. Protoc. Chem. Biol.* **2013**, *5*, 171–194.

[12] G.E. Crooks, G. Hon, J.M. Chandonia, S.E. Brenner, *Genome Res.* **2004**, 14, 1188–1190.

[13] D. Fujiwara, H. Kitada, M. Oguri, T. Nishihara, M. Michigami, K. Shiraishi, E. Yuba E, I. Nakase, H. Im, S. Cho, J.Y. Joun, S. Kodama, K. Kono, S. Ham, I. Fujii, *Angew. Chem. Int. Ed.* **2016**, 55, 10612–10615.

[14] C. N. Pace, F. Vajdos, L. Fee, G. Grimsley, T. Gray, *Protein Sci.* **1995**, *4*, 2411–2423.

[15] H. Nordin, M. Jungnelius, R. Karlsson, O.P. Karlsson, *Anal. Biochem.* **2005**, *340*. 359–368.

[16] S. Afshar, T. Asai, S.L. Morrison, *Mol. Cancer Ther.* **2009**, 8, 185–193.

[17] K. Anderson, Z. Lai, O.B. McDonald, J.D. Stuart, E.N. Nartey, M.A. Hardwicke, K. Newlander, D. Dhanak, J. Adams, D. Patrick, R.A. Copeland, P.J. Tummino, J. Yang, *Biochem. J.* **2009**, *420*, 259–265.

[18] P.R. Sheth, L. Ramanathan, A. Ranchod, A.D. Basso, D. Barrett, J. Zhao, K. Gray, Y.H. Liu, R. Zhang, H.V. Le, *Arch. Biochem. Biophys.* **2010**, *503*, 191–201.

[19] K.S. Oh, B.K. Oh, C.H. Park, J. Mun, S.H. Won, B.H. Lee, *Biol. Pharm. Bull.* **2012**, *35*, 1281–1286.

[20] E.R. Sharlow, K.V. Giridhar, C.R. LaValle, J. Chen, S. Leimgruber, R. Barrett, K. Bravo-Altamirano, P. Wipf, J.S. Lazo, Q.J. Wang, *J. Biol. Chem.* **2008**, *283*, 33516–33526.

[21] E.A. Harrington, D. Bebbington, J. Moore, R.K. Rasmussen, A.O. Ajose-Adeogun, T. Nakayama, J.A. Graham, C. Demur, T. Hercend, A. Diu-Hercend, M. Su, J.M. Golec, K.M. Miller, *Nat. Med.* **2004**, *10*, 262–267.
